# Therapeutic Interventions to Mitigate Mitochondrial Dysfunction and Oxidative Stress–Induced Damage in Patients with Bipolar Disorder

**DOI:** 10.3390/ijms23031844

**Published:** 2022-02-06

**Authors:** Sahithi Madireddy, Samskruthi Madireddy

**Affiliations:** 1 Massachusetts Institute of Technology, Cambridge, MA 02139, USA; 2 Department of Neuroscience, Johns Hopkins University, Baltimore, MD 21218, USA; smadire2@jhu.edu

**Keywords:** bipolar disorder, oxidative stress, mitochondrial dysfunctions, immune system dysregulation, nutrients, antipsychotics, anti-inflammatory agents, lithium therapy, light therapy

## Abstract

Bipolar disorder (BD) is characterized by mood changes, including recurrent manic, hypomanic, and depressive episodes, which may involve mixed symptoms. Despite the progress in neurobiological research, the pathophysiology of BD has not been extensively described to date. Progress in the understanding of the neurobiology driving BD could help facilitate the discovery of therapeutic targets and biomarkers for its early detection. Oxidative stress (OS), which damages biomolecules and causes mitochondrial and dopamine system dysfunctions, is a persistent finding in patients with BD. Inflammation and immune dysfunction might also play a role in BD pathophysiology. Specific nutrient supplements (nutraceuticals) may target neurobiological pathways suggested to be perturbed in BD, such as inflammation, mitochondrial dysfunction, and OS. Consequently, nutraceuticals may be used in the adjunctive treatment of BD. This paper summarizes the possible roles of OS, mitochondrial dysfunction, and immune system dysregulation in the onset of BD. It then discusses OS-mitigating strategies that may serve as therapeutic interventions for BD. It also analyzes the relationship between diet and BD as well as the use of nutritional interventions in the treatment of BD. In addition, it addresses the use of lithium therapy; novel antipsychotic agents, including clozapine, olanzapine, risperidone, cariprazine, and quetiapine; and anti-inflammatory agents to treat BD. Furthermore, it reviews the efficacy of the most used therapies for BD, such as cognitive–behavioral therapy, bright light therapy, imagery-focused cognitive therapy, and electroconvulsive therapy. A better understanding of the roles of OS, mitochondrial dysfunction, and inflammation in the pathogenesis of bipolar disorder, along with a stronger elucidation of the therapeutic functions of antioxidants, antipsychotics, anti-inflammatory agents, lithium therapy, and light therapies, may lead to improved strategies for the treatment and prevention of bipolar disorder.

## 1. Introduction

Bipolar disorder (BD) is a chronic mental illness characterized by an alternation between mania or hypomania and depression [1,2,3,4]. It is often associated with impaired functionality [5,6]. Neurotransmitter imbalance, oxidative stress (OS), and genetic causes are some of the factors that have been linked to the pathophysiology of BD [7,8]. A consistent finding in BD is the presence of OS, which makes biomolecules susceptible to oxidative and nitrosative damage [9]. Dopamine (DA) levels are notably increased during mania, and DA produces reactive oxygen species (ROS) and quinones that can proceed to oxidize proteins [9]. The overproduction of ROS and reactive nitrogen species, along with impaired maintenance of balance by antioxidant systems, can result in damage to lipids, proteins, DNA, and RNA [9,10]. In addition, the presence of ROS/reactive nitrogen species in mitochondria leads to oxidation of mitochondrial DNA (mtDNA), proteins, and lipids [10,11]. Inflammation and immune dysfunction may be involved in BD pathophysiology [12,13,14,15,16,17]. This review focuses on the roles of ROS and ROS-induced oxidative damage, mitochondrial dysfunction, DNA damage, and DA system dysregulation, and immune dysfunction in the pathophysiology of BD. It also presents an overview of potential biomarkers, including lipid peroxidation, thiobarbituric acid reactive substances (TBARSs), and brain-derived neurotrophic factor (BDNF), in patients with BD.

Diet influences several processes that are dysregulated in BD, including monoaminergic activity, inflammation and immune processes, OS, mitochondrial function, and neuroprogression. Therefore, in this review, we also discuss the possible use of nutrients, including vitamin D, copper, folic acid, and polyunsaturated fatty acids (PUFAs), in the treatment of patients with BD. In addition to nutraceutical approaches, we further discuss the use of anti-inflammatory agents and novel antipsychotic agents, such as clozapine, olanzapine, risperidone, cariprazine, and quetiapine, as alternatives to lithium or conventional antipsychotic agents. Furthermore, we assess the use of therapies, such as cognitive–behavioral therapy (CBT), bright light therapy (BLT), imagery-focused cognitive therapy (ImCT), and electroconvulsive therapy (ECT), to treat BD.

## 2. BD

BD is one of the most debilitating psychiatric disorders, and it involves abnormal neuroplasticity [18,19,20,21,22,23,24]. It is defined by disruptive depressive and manic or hypomanic episodes [25,26,27,28,29,30,31,32]. The two main categories of BD are type I (BD-I) and type II (BD-II) [33,34]. BD-I is characterized by at least one manic episode, involving increased activity, libido, and grandiose thinking, followed by a hypomanic or depressive episode [35,36,37,38]. On the other hand, BD-II includes at least one hypomanic and depressive episode but does not present with manic episodes [39,40]. Despite the availability of current pharmacological and psychosocial treatments, BD can increase the risk of substance abuse, suicide, and mortality from comorbidities [41,42,43,44,45,46,47,48,49,50,51].

BD is a complex disorder involving environmental, social, and genetic factors [52,53]. Biological processes in the pathophysiology of BD include perturbations of brain development, chronobiology, and neuroplasticity; defective apoptotic, inflammatory, neurotrophic, neurotransmitter, and calcium signaling; oxidative and nitrosative stress; endoplasmic reticulum stress; as well as mitochondrial dysfunction [54,55,56,57,58,59,60,61,62,63]. As the parietal lobe supports cognition, attention, and memory, it is greatly involved in the progression of BD [39,64]. Some patients with BD exhibit neuroprogression [65,66,67], which is characterized by progressive changes in neuroanatomy, including decreased hippocampal volume [68], increased lateral ventricle size [69], and decreased cortical thickness [70]. These neuroanatomical changes are associated with functional impairment. [65,66,67,68,69,70].

## 3. OS

An imbalance between antioxidative and oxidative processes leads to OS, which can produce ROS and free radicals that proceed to damage cellular components [71,72,73]. OS leads to not only mutations in mtDNA and damage to the electron transport chain but also changes in membrane permeability, mitochondrial defense mechanisms, and calcium levels [74]. The central nervous system is especially vulnerable to OS because the brain uses large amounts of oxygen, which increases the production of free radicals and ROS [75,76].

ROS are free radicals that contain oxygen; they form as a byproduct of electron transport [77]. They are created by the premature discharge of electrons from complexes near the start of the electron transport chain [77]. This process leads to the formation of superoxide anions (O_2_•^−^). Superoxide dismutase (SOD) turns superoxide radicals to hydrogen peroxide (H_2_O_2_); catalase (CAT) and glutathione peroxidase (GPX) convert H_2_O_2_ to water and oxygen [78,79,80,81,82,83]. Under normal conditions, 1% to 5% of the oxygen consumed by a cell forms ROS [84]; by contrast, under conditions of mitochondrial insult, such as mitochondrial toxin exposure or excess calcium, oxygen conversion to ROS is increased [77]. Excessive intake of nutrients can also produce large quantities of ROS because the electron transport chain might become overloaded with electrons, causing their promiscuous release [85,86]. Failure to eliminate ROS can lead to oxidative injury to the cell, including peroxidation of lipids, DNA, and proteins (e.g., receptors and enzymes) [87,88,89]. The iron–sulfur clusters involved in electron transport in complexes I and III are most vulnerable to oxidative damage [86].

**Figure 1:**
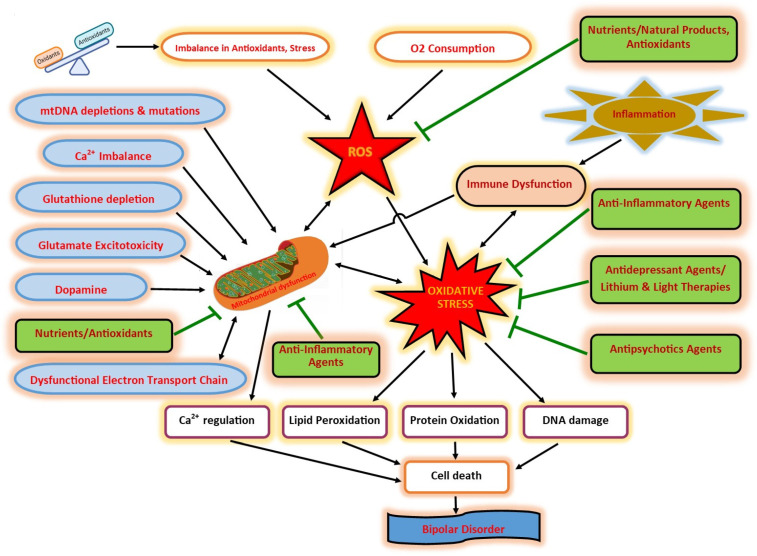
The potential interactions between BD, OS, mitochondrial dysfunction, immune dysfunction, antioxidants, antidepressant agents, anti-inflammatory agents, antipsychotic agents, and light therapies.

DA is another factor that may contribute to OS [90]. Through spontaneous auto-oxidation or with enzyme catalysis, DA can easily be oxidized to form quinones and ROS [91]. This outcome produces H_2_O_2_, which can react with transition metals to create toxic hydroxyl radicals [91]. In addition, the electrophilic DA quinones formed by auto-oxidation may potentially react with thiol compounds [91]. Both ROS and quinones may proceed to damage cellular components. Moreover, the products of DA oxidation inhibit the activity of cysteine-rich proteins, such as DA transporter (DAT) [91]. Figure 1 illustrates the potential interactions between BD, OS, mitochondrial dysfunction, immune dysfunction, antioxidants, antidepressant agents, anti-inflammatory agents, antipsychotic agents, and light therapies.

## 4. Oxidative Damage in BD

OS has been posited to play a role in the development of several psychiatric disorders, including BD [92,93,94]. For instance, two recent studies found increased OS in BD [93,95]. Moreover, postmortem samples of the blood and brain revealed that younger patients with BD consistently show damage from OS [96,97,98,99,100,101,102]. Alterations in neuroplasticity, signaling, and neurotransmitter uptake by increased OS may be involved in the pathogenesis of these dysfunctions [103,104]. These harmful changes may be partially a consequence of increased lipid peroxidation in membranes, proteins, and DNA due to increased OS [105,106].

Such biomarkers as protein oxidation, 8-hydroxydeoxyguanosine, and lipid peroxidation can indicate the extent of oxidative damage [107,108]. Increased lipid peroxidation has been observed in the prefrontal cortex and anterior cingulate cortex of patients with BD [109]. Moreover, in their meta-analysis, Brown et al. reported increased lipid peroxidation and damage to DNA and RNA in BD [110]. Another meta-analysis of 27 articles, encompassing 971 patients with BD and 886 healthy controls, found significantly higher lipid peroxidation, nitric oxide, and damage to DNA and RNA in patients with BD compared with controls [110]. These findings support the potential role of OS in BD pathogenesis, particularly through its effects on lipids, DNA, and RNA [110,111].

Other measures of OS are total antioxidant status (TAS), total oxidant status (TOS), and OS index, which indicates the balance between oxidation and antioxidation [107]. The total activity of antioxidants is demonstrated by TAS, whereas that of ROS is demonstrated by TOS. The OS index is determined by the ratio of TOS to TAS, and this reflects the overall OS level [107]. One study conducted with 94 BD patients and 41 healthy controls reported higher TAS, TOS, and OS index levels in the BD patients compared with the controls [73]. It also investigated how differences in these measures depended on the type of BD. TAS was negatively correlated with the number of episodes a patient experienced, especially in BD-I. In addition, TOS seemed to be higher in BD-I than in BD-II [73]. The increased TAS in BD patients in said study may be a response to increased levels of oxidation. The same observation was reported by Yumru et al., who found higher TAS levels in the serum of BD patients who were euthymic compared with healthy controls [73].

TBARSs, carbonyl proteins, malondialdehyde (MDA), and nitrites have also been used as peripheral markers of OS. Additional antioxidant markers include total antioxidative capacity; measures of antioxidants (glutathione, zinc, and uric acid); as well as levels of SOD, GPX, and glutathione *S*-transferase [94,112]. A study of 113 BD patients who were euthymic and 78 controls found decreased TBARS, MDA, and total antioxidative capacity levels in the BD patients compared with the controls [94]. It also found decreased antioxidant and OS markers; however, many other studies have reported the opposite finding. For example, some studies observed increased TBARS levels during mania, depression, and remission [113], implying that OS may be stable throughout the course of BD [88]. Another study corroborated this finding of increased serum TBARS levels in BD patients during mania, depression, and euthymia [114]. Moreover, Sowa-Kućma et al. found a significant positive association between higher TBARS level and severity of BD, including the risk of suicidality [115].

As specified earlier, a frequent finding in patients with BD is the increase in antioxidant marker levels [116], which is likely part of a compensatory response to prevent oxidative damage. Yumru et al. found that serum SOD (an antioxidant marker) was significantly elevated in patients with depression and mania compared with controls [73]. Furthermore, Andreazza et al. reported a significant increase in glutathione *S*-transferase and glutathione reductase in late-stage BD patients [117]. A meta-analysis of 44 studies, covering 1979 BD patients and 1788 controls, found that BD was correlated with elevated CAT, glutathione *S*-transferase, nitrite, TBARS, MDA, and uric acid levels [112]. In addition, BD was correlated with a decrease in glutathione; changes in GPX, SOD, and zinc levels were not observed [112]. Uric acid and TBARSs were increased in patients who were manic; moreover, TBARSs were increased in patients during depression, and uric acid was increased in patients during euthymia [112].

Other researchers have also examined how OS and antioxidant markers vary over the phases of BD. Some of them observed that TBARS and SOD levels in BD patients who were experiencing manic and depressive episodes were higher than those in BD patients who were euthymic and those in controls [95,118]. Similarly, Kunz et al. found that SOD activity was only elevated in acute BD and did not significantly differ between controls and BD patients who were euthymic [118]. As mentioned previously, elevated SOD levels may be part of a compensatory response to excess ROS production during an acute phase of BD. This overproduction would mobilize antioxidant defenses and systems of neuronal plasticity [81]. Consistent with the findings of Kunz et al., Andreazza et al. [119] and Machado-Vierra et al. [95] reported higher SOD levels and higher CAT and SOD levels, respectively, in BD patients who were manic compared with controls. However, Gergerlioglu et al. found that BD patients had decreased SOD levels during manic episodes [120].

Several key findings regarding how OS mechanisms can contribute to BD have been reported. Excess ROS can damage mitochondria through mutations to mtDNA, thereby damaging the electron transport chain and altering membrane permeability [74,121,122]. ROS overproduction can also damage proteins that regulate calcium, such as gated calcium channels, calcium ion (Ca^2+^)–ATP synthases, and proteins in the electron transport chain [74]. This outcome disrupts calcium homeostasis, leading to elevated calcium [74]. SOD and glutathione are key components of the antioxidant defense against OS [72,123]. Therefore, increased SOD activity may be seen as a mechanism of mitigating increased oxidative damage in BD [81].

On the other hand, increased SOD and CAT levels could also cause increased H_2_O_2_ production, which may contribute to neural damage through oxidation of lipids and proteins [124,125]. Levels of uric acid in BD patients undergoing mania have been found to be higher than those in BD patients undergoing depression but not those in BD patients undergoing euthymia [112]. Differences in TBARS levels between mania and depression have not been observed [112]. Compared with healthy controls, BD patients who were not taking medication have been reported to exhibit increased SOD levels and decreased GPX levels. BD patients and healthy controls have been found to demonstrate comparable SOD and GPX levels after treatment. Overall, BD is correlated with OS, uric acid/TBARS levels are increased in specific phases, and treatment may restore SOD and GPX to normal levels [112,126].

## 5. Mitochondrial Dysfunction

Mitochondria are responsible for energy production and providing substrates for cell growth. In addition, they are involved in cell resilience and oxidative/nitrosative stress [127,128,129,130,131]. Mitochondria in the brain are important for influencing neural activity, neural plasticity, and behavioral adaptation through their effects on long-term potentiation [132,133,134,135,136]. Mitochondrial dysfunction has been shown to be crucial in BD [137,138,139,140,141,142,143]. BD may include underlying mitochondrial dysfunction based on observations of decreased cellular respiration, altered mitochondrial structure, mtDNA mutations, and decreased production of proteins involved in respiration [54,87]. Failure of respiration is further evidenced by a decrease in pH and decreased presence of compounds containing high-energy phosphates in the brain [54,87].

Moreover, mitochondrial disorders may lead to psychotic, affective, and cognitive symptoms [54,87]. Genetic, postmortem, and molecular studies have indicated that mitochondrial dysregulation could lead to the nervous system impairment observed in BD [144]. Some patients with BD have been found to exhibit deterioration in mitochondrial quality control mechanisms [141]. In addition, postmortem analyses of the brains of patients with BD have found decreased expression of genes involved in the electron transport chain [145]. As noted previously, BD is also associated with mutations and polymorphisms of mtDNA [146,147,148,149,150]. Because it lacks the protection of histones, mtDNA is especially vulnerable to mutations from oxidative damage [53]. Mitochondrial dysfunction increases ROS generation, which leads to greater OS [151]. Consistent with this reasoning, OS markers have been reported to be increased in the brains of BD patients analyzed postmortem as well as in the blood of BD patients [91,152]. Mitochondrial dysfunction may also play a role in the altered calcium signaling observed in BD [153,154].

## 6. DNA Damage

OS can cause DNA damage, which some studies on patients with BD have observed [74,155,156]. A significant association between DNA damage and BD as well as associations between DNA damage and the severity of BD and depressive symptoms have been reported [157]. In particular, Andreazza et al. showed a positive correlation between DNA damage and the severity of manic and depressive symptoms [113]. With the use of comet assay to evaluate DNA damage, they observed increased DNA damage in BD patients compared with controls [113]. Another study characterized the OS profile of monozygotic twins who were experiencing manic episodes [81]. Compared with healthy twins, bipolar twins had elevated SOD, TBARS, and DNA damage [81]. The bipolar twins also had decreased CAT levels. After treatment with mood stabilizers, TBARSs and SOD in the bipolar twins returned to normal levels; however, CAT levels and DNA damage were still abnormal 6 weeks after the treatment started [93]. In a rat model of mania, amphetamine administration increased DNA damage in the blood and hippocampus [81], and DNA damage was positively correlated with lipid peroxidation [89].

## 7. BDNF

BDNF is important for the development, plasticity, and survival of neurons [75,158,159]. Six meta-analyses found decreased BDNF levels in BD patients compared with both healthy individuals and patients with unipolar depression [52,160,161,162,163]. Lowered BDNF levels were observed with both mania and depression in BD [52,160,161,162,163]. Patients with BD may have abnormal plasma levels of other neurotrophins as well [75]. The link between higher levels of OS and decreased BDNF levels has been well demonstrated in BD [54,81].

In addition, a connection between mitochondrial complex I dysfunction, ROS production, and decreased BDNF level has been established [164]. A study of 59 BD patients and 26 healthy controls examined the association of BDNF levels with antioxidant defenses [165]. It found that the peripheral BDNF level in BD was correlated with antioxidant enzyme activity [165]. Another study also found a negative correlation between TBARS and BDNF levels in patients with BD, implying that a modified oxidative status might lead to decreased BDNF levels [166]. A meta-analysis demonstrated substantial reductions in peripheral BDNF levels in manic and depressive episodes of BD [163]. A similar meta-analysis consisting of 35 studies and 3798 research participants reported that BD patients exhibited lower peripheral BDNF levels compared with healthy controls [52]. Another study measured mtDNA copy number in leukocytes, plasma BDNF level, and antioxidant enzyme activity in 97 BD patients and 31 healthy controls [158]. BDNF level, mtDNA copy number, and GPX activity in the BD patients were significantly lower than those in the controls [158]. Other researchers have examined the association between plasma BDNF level and the functions of GPX and SOD in BD patients and healthy controls [165]. Peripheral BDNF level in BD and antioxidant enzyme activity had a robust correlation regulated by metabolic comorbidities [165]. Overall, these findings suggest that a lowered BDNF level may be a component of the pathophysiology of BD [52,160].

## 8. Roles of DA and DAT in BD

DAT is critical to the dopaminergic system due to its function in DA reuptake, causing its removal from the synaptic cleft [91]. Recent studies have highlighted a possible link between DAT and mania [91]. The enhanced dopaminergic transmission accompanying mania can contribute to OS in patients with BD [89,167]. Elevated DA causes a significant increase in ROS production and mitochondrial dysfunction, which may further damage DNA and cause cell death [91,168]. OS can induce posttranslational modifications of the DAT, which would decrease DA reuptake [91]. Because patients with BD have lower levels of antioxidant enzymes [169], they may be highly vulnerable to DAT oxidation [170]. Toxicity from increased DA can kill dopaminergic neurons, potentially resulting in the depressive phase of BD [167]. Notably, pharmaceutical therapies for BD, including *N*-acetylcysteine and lithium, may protect against OS and DA toxicity [171,172,173], pointing to the possibility of preventing the vicious cycle of DA inhibiting the DAT [91]. Therefore, understanding DA dysregulation in BD will help determine the pathophysiology of BD and may assist in the development of novel therapeutic agents to augment treatment [91].

## 9. Immune Dysfunction

BD development may be triggered by immune system dysregulation [16,174,175,176] such as acute-phase protein and cytokine alterations, which can cause BD through neurotransmitter- and neuropeptide-related effects [177,178]. Proinflammatory cytokines include interleukin (IL)-6 and tumor necrosis factor-alpha (TNF-α), whereas anti-inflammatory cytokines include IL-10 and IL-4, which can prevent immune system activation [179,180,181]. Notably, high proinflammatory cytokine levels have been consistently observed in patients with BD, especially during acute episodes [177,180,182,183,184]. Moreover, patients with BD exhibit reduced anti-inflammatory cytokine levels during the manic phase [185,186,187,188]. Even genetic studies have indicated that a multitude of genes that partake in various neuroimmunological and inflammatory pathways are either up or downregulated in BD. Genetic studies have suggested that the presence of BD correlates with differential upregulation or downregulation of several genes involved in inflammatory and neuro-immunological pathways [189]. Circulating mtDNA and inflammation level, indicated by plasma cytokine (GM-CSF, IL-2, IL-4, and IL-6) measurements, were correlated in the patients [190]. In another study, patients with BD generally showed increased proinflammatory cytokine levels [191]. In addition, depressed patients displayed reduced plasma anti-inflammatory cytokine and increased TNF-α levels, suggested to play a potential role in treatment resistance [191,192,193]. In another study investigating the relationships between OS, cytokines, and circadian preferences, plasma IL-10, IL-6, and TNH-α were measured [194]. Among BD patients, there was a greater change in circadian rhythms than in controls and patients with major depressive disorder [194]. Those BD patients who had reversed day/night cycles also had lower serum IL-6, IL-10, and TBARS. This suggests that oxidative stress may affect immune function and may be correlated with CNS functions in a subset of BD patients [194]. A 2021 meta-analysis described increased IL-6, TNF-α, and C-reactive protein (CRP) levels in patients with BD, with particularly increased TNF-α and CRP levels during manic and depressive episodes [195]. Another meta-analysis found elevated TNF-α, IL-4, and soluble IL-2 and IL-6 receptor levels in patients with BD compared to healthy controls [196]. In addition, certain pieces of evidence suggest that antidepressants could reduce systemic inflammation, even though the anti-inflammatory effects of antidepressants are yet to be evaluated fully [197]. Moreover, the immune system could contribute to BD pathology through the regulation of the hypothalamic–pituitary–adrenal (HPA) axis [177]. Proinflammatory cytokines increase HPA axis activity, resulting in increased systemic cortisol levels [198].

## 10. Calcium Signaling Pathways

One hypothesis for BD states that mtDNA mutations or mitochondrial RNA deletions lead to impaired mitochondrial regulation of calcium, causing BD symptoms [54,199]. Consistent with this hypothesis, calcium levels in peripheral cells of patients with BD have been found to be higher than normal [77,200,201,202]. A persistent increase in intracellular calcium can cause neurons to undergo degeneration and die [203]. Excess calcium in mitochondria induces mitochondrial permeability transition, after which the mitochondria swell and the outer mitochondrial membrane ruptures [204]. Furthermore, ROS production in mitochondria stimulates the uptake of Ca^2+^ and enhances membrane permeability, leading to cytochrome *c* release and the initiation of apoptosis [203,205]. Mitochondrial permeability transition further hampers oxidative phosphorylation, inhibits citric acid cycle enzymes, reduces ATP synthesis, increases ROS generation, as well as increases the release of calcium and apoptogenic factors to the cytosol [54,206,207]. Increased calcium concentrations also result in an altered mitochondrial potential and the formation of superoxide ion radicals [204], thus contributing to a vicious cycle [74].

## 11. Nutrients, Vitamins, and Micronutrients in BD

Consumption of nutrients are beneficial to brain health and its functioning [208]. Diet can affect a range of processes that are altered in BD, such as monoaminergic activity, mitochondrial activity, inflammation, OS, and neuroprogression [209,210,211]. Oxidants may be crucial to psychiatric disorders as they are linked to membrane-related pathology in the central nervous system [212,213]. Certain oxidants can cause adverse increases in other metabolites, which can lead to specific psychiatric symptoms [73]. As previously noted, BD is characterized by lipid peroxidation and changes in antioxidant enzymes [214]. Consequently, antioxidant compounds may improve symptoms and may be explored as an adjunctive therapy. For instance, minocycline, an antibiotic, seems to have neuroprotective effects through its antioxidant activity, a mechanism that is also applicable to the pathophysiology and treatment of BD [215,216]. Poor nutrition is often associated with OS and inflammation, which can impact the immune system [217]. Early studies suggest that anti-inflammatory agents are likely to be beneficial for patients with BD exhibiting immune dysregulation [15].

### 11.1. Vitamin D

A study of 118 BD patients found a vitamin D deficiency rate 4.7× greater than that in the general population [218]. In a cross-sectional case–control study, Naifar et al. measured 25-hydroxy vitamin D in the plasma of patients with acute decompensation of BD relative to healthy controls [219]. In contrast to the prior study, their analysis discovered significantly higher levels of 25-hydroxy vitamin D in the BD patients compared with the healthy controls [219]. The study revealed that an increase in 25-hydroxy vitamin D production is correlated with acute decompensation of BD. Vitamin D supplementation correlates with a decline in manic and depressive symptoms, but further studies on the efficacy of specific doses are needed to corroborate these data [220,221,222]. Vitamin D has also been shown to exhibit anti-inflammatory effects [217].

### 11.2. Folic Acid

Folic acid and folates are considered useful for treating depressive symptoms [223] due to their role in neurotransmitter synthesis and DNA methylation [224]. As reported in multiple studies, individuals who are depressed have lower concentrations of plasma and erythrocyte folate compared with both healthy people and people with other psychiatric disorders [225]. Reduced levels of folate correlate with poor responsiveness to antidepressant medication [226]. One study examined adding 200 μg/d of folic acid to lithium treatment in BD, with the authors ultimately suggesting that folic acid supplementation may be used during maintenance therapy [226]. Behzadi et al. reported positive outcomes from the inclusion of folic acid along with valproates in the treatment of mania [227]. Moreover, recent studies encompassing a group of 10 BD-I patients undergoing depression have shown the advantageous effects of augmenting standard treatment with levomefolic acid [228]. Intake of folic acid as levomefolic acid may be more effective because levomefolic acid is more bioavailable [228].

### 11.3. Magnesium and Copper

Several reports have identified variations in the blood magnesium concentration of patients with BD [229]. One study highlighted significantly increased serum magnesium concentrations in patients with BD during mania, hypomania, and depression. However, during remission, serum magnesium returned to normal levels and did not differ from the levels in healthy controls [229]. Another study measured magnesium concentration in 129 BD patients (23 exhibiting mania, 58 exhibiting depression, and 48 in remission) along with 50 healthy controls [229]. It found that the BD patients undergoing a depressive, manic, or hypomanic episode had significantly higher serum magnesium concentrations compared with the healthy controls [229]. The serum magnesium levels of the patients in remission were unaltered compared with those of the controls. These results suggest that serum magnesium may be used as a potential marker of the pathophysiological alterations accompanying acute BD [229]. In addition, several reports have noted altered serum copper levels in patients with BD [230]. A study of 133 BD patients (23 exhibiting mania/hypomania, 61 exhibiting depression, and 49 in remission) showed significantly increased levels of serum copper among patients in stage I compared with patients in advanced stages of the disorder [230].

### 11.4. PUFAs

Omega-3 PUFAs, such as eicosapentaenoic acid and docosahexaenoic acid (DHA), are critical to the development and activities of the brain, including neuronal migration, maturation, formation of synapses, neuronal plasticity, and synaptic transmission [226]. Alterations in PUFA levels have been suggested to be present in BD [231,232]. An analysis of six studies including a total of 118 BD-I patients and 147 healthy controls showed deficits in erythrocyte DHA and decreased eicosapentaenoic acid in the BD-I patients [232]. Omega-3 PUFAs have been suggested as possible therapeutic supplements for a variety of illnesses, such as cancer, diabetes, arteriosclerosis, hypertension, arthritis, psychiatric disorders, dementia, and autoimmune diseases [233]. More specifically, study results for the use of omega-3 PUFAs in BD have been encouraging [234,235,236,237]. The increase in BDNF levels caused by omega-3 PUFAs has been proposed to account for how omega-3 PUFAs may enhance the outcomes of BD [235]. In addition, recent research indicates that anti-inflammatory medication might contribute to mood disorder treatment [21]. Omega-3 PUFAs, naturally occurring anti-inflammatory agents, are found to be well-tolerated [208,238,239]. In a randomized controlled trial (RCT), omega-3 fatty acids displayed a significant antidepressant effect in subjects with high inflammatory marker levels [240].

Multiple epidemiological and experimental studies have considered the association between dietary intake/supplementation of PUFAs and incidence or severity of depression [241]. Some research studies have also proposed that increased dietary PUFA consumption in patients with BD is beneficial [242]. Daily administration of 1 to 2 g of eicosapentaenoic acid has been found to reduce depressive symptoms, including those in patients with BD-I [243]. Another study examined the effectiveness of prophylactic administration of omega-3 PUFAs in BD: Eighty patients with BD were randomized such that 40 received placebo and the other 40 received 1 g of eicosapentaenoic acid as well as 1 g of DHA as adjunctive therapy for 52 weeks [244]. The study determined that omega-3 PUFA administration had a prophylactic effect in the patients. A double-blind randomized trial assessed daily DHA supplementation at 1250 mg versus placebo for 12 weeks in 31 BD patients who were euthymic and 15 healthy controls [245]. In contrast to the above-mentioned results, the trial found improved cognitive function based on performance in emotion inhibition only in healthy controls who received DHA for 12 weeks. This finding suggests that DHA supplementation may be effective in increasing cognitive performance, but further research on this topic is required [245].

## 12. Anti-Inflammatory Agents

In addition to omega-3 fatty acids and vitamin D, other nutrients with reported anti-inflammatory and antioxidant effects include vitamin A, vitamin C, and phytochemicals such as polyphenols and carotenoids [246,247,248]. Vitamin C is considered an antioxidant because it quenches free radicals while being oxidized into dehydroascorbic acid [248]. Moreover, it has been shown to act on neutrophils to induce phagocytosis, ROS generation, and migration to the infection site [249,250]. Omega-3 fatty acids; vitamins A, C, and D; polyphenols; carotenoids; and other anti-inflammatory compounds might contribute to the homeostatic regulation of OS and inflammation, both under normal conditions and during infection. Clinical trials involving patients with BD have demonstrated encouraging results for a diverse group of anti-inflammatory agents [251]. When aspirin, celecoxib, infliximab, *N*-acetylcysteine (NAC), omega-3 fatty acids, and pioglitazone were administered as adjuvant therapy, they were found to be effective in reducing BD-related depression [251]. Evidence has particularly supported the use of NAC as an adjuvant therapeutic agent for BD-related depression [252]. In an RCT involving 75 patients with BD, adjunctively administered NAC significantly reduced depression severity after 24 weeks compared to conventional treatment alone [253]. Of note, valproate also exhibited anti-inflammatory effects both systemically and in the CNS in encephalomyelitis rat models [254]. Moreover, valproic acid was found to reduce proinflammatory cytokine production in controls [255].

### Lithium Therapy

Lithium, the first-line drug treatment for BD, both for bipolar depression [256,257,258,259,260,261] and for mixed episodes [262,263,264,265,266,267,268,269,270,271,272,273,274] has numerous neuroprotective, neurotrophic, and neuroplastic effects [275,276,277,278]. In addition to being the mood stabilizer conventionally used in ameliorating the pathophysiology of BD, lithium has some antidepressant activity [279,280,281,282]. The therapeutic effects of lithium have been hypothesized to be partially linked to its antioxidant capabilities. One study examined 29 BD patients in a depressive episode who were treated with lithium for 6 weeks as well as 28 controls [145]. Plasma TBARS levels as well as SOD, CAT, and GPX activities were measured at baseline and 6 weeks in both groups [145]. Lithium administration only caused a decrease in TBARS and SOD levels; this was most evident in BD-II [106,145]. TBARS levels were significantly lower after 6 weeks in patients who responded to lithium compared with those who did not [95,145].

A similar study showed significantly lower TBARS and SOD:CAT levels in BD patients [283] who were administered lithium but not in healthy people given lithium [155,284]. Short-term lithium treatment has been shown to lower SOD:CAT and TBARS levels in patients with BD who were experiencing mania as well [95]. These outcomes were corroborated by a follow-up study showing a reduction in SOD and TBARS levels in patients with BD after 6 weeks of lithium therapy [285]. The study also found that lithium responders exhibited significantly lower TBARS levels compared with nonresponders [106].

Taken together, these findings support the role of OS in the pathophysiology of BD, in addition to the role of the antioxidant activity of lithium in providing effective clinical intervention [286,287,288,289]. Moreover, lithium reduces proapoptotic activities and increases the level of neuroprotective proteins, including Bcl-2 [81,290,291]. These mechanisms may contribute to the neuroprotective action of the drug. Lithium reportedly exhibits anti-inflammatory activity through the inhibition of IL-1B, TNF-α, and cyclooxygenase-2 synthesis and stimulation of IL-2 and IL-10 production [16,292,293,294]. There have also been reports on lithium’s proinflammatory effects, such as the enhancement of IL-4, IL-6, and other inflammatory cytokine synthesis [208,295,296]. Through its anti-inflammatory effects, lithium was found to decrease proinflammatory cytokine levels and alleviate manic behavior in a mouse model of mania [297]. Two recent preclinical studies reported on the neuroprotective effects of lithium. In a study focusing on rat glia, lithium pretreatment reduced TNF-α, IL-1β, NO, and prostaglandin E_2_ secretion in response to lipopolysaccharide-induced inflammation [298,299]. In another study using a rat model of intracerebral hemorrhage, lithium prevented perihematomal cell death and reduced COX-2 expression and reactive microglia number [298,300]. In addition to regulating apoptosis, lithium is known to protect against excitotoxicity [301,302] as well as to increase BDNF and intracellular calcium levels [145]. It causes an increase in neuroplasticity, which mitigates the reduction in gray and white matter observed in BD [301] and may ameliorate the visuospatial asymmetry produced by BD [303]. Lithium therapy has demonstrated frontal cortex enhanced electron transport chain complexes I, II, and III activity in the postmortem brains of patients with BD [304]. In addition, de Sousa et al. observed the ability of lithium to improve the activity of electron transport chain complex I in the leukocytes of patients with BD to an extent dependent on the plasma lithium level [305]. By affecting a range of biological processes, lithium has proven to be a potent treatment for BD [306,307,308,309,310,311,312].

## 13. Antipsychotic Agents

Antipsychotic agents are effective in managing the symptoms of mania by blocking DA D_2_ receptors [91,313]. Therefore, people with BD-I who undergo manic episodes are more likely to be prescribed antipsychotic agents [314,315]. A recent meta-analysis showed the efficacy of typical and atypical antipsychotic agents in the treatment of mania, suggesting that disrupted signaling of DA may contribute to the presentation of manic symptoms in patients with BD [316,317]. Atypical antipsychotic agents, including olanzapine, clozapine, risperidone, quetiapine, aripiprazole, cariprazine, and ziprasidone, are classified as first-line medications for psychotic depression [314,318,319,320,321]. They are the preferred antipsychotic agents for long-term maintenance therapy [318,319,320,321].

### 13.1. Clozapine

Some patients with BD, including those who respond poorly to mood stabilizers and typical antipsychotic agents or who exhibit rapid cycling, may benefit from the use of clozapine [314,322]. Especially among older adults, clozapine may be beneficial in the treatment of BD and psychotic disorders [323]. Several research studies have shown that the efficacy of clozapine is increased during the manic phase of BD, as depicted in a study where patients with BD who were in a manic/mixed state responded better than did those who were in a depressed state [314]. The effectiveness of clozapine in the maintenance treatment of BD has been described in four open-label studies, of which three with a prospective study design specifically explored its effectiveness in mania [314].

In one study assessing the clinical benefit and adverse effects of clozapine, 100 and 102 patients with BD were administered clozapine and other antipsychotic agents, respectively [324]. Clozapine was found to have equivalent efficacy relative to the other antipsychotic agents for mania, and it outperformed them for treatment-resistant BD (TRBD) [324]. Fifteen clinical trials with 1044 patients in total were designed to evaluate the use of clozapine for TRBD [325]. Although the existing data are limited, they support the use of clozapine as a potent and safe treatment for TRBD [325]. In a mirror image study, 62 patients with BD who were in remission initially received clozapine treatment; of those patients, 25 were transferred to another antipsychotic treatment after a change in drug reimbursement, whereas 37 continued receiving clozapine [326]. The study indicated that a shift from clozapine to another antipsychotic agent might increase the likelihood of recurrence for BD patients in remission [326]. In addition, a case study found that 3 BD patients who had suicidal ideation benefited from the addition of clozapine to their treatment regimen, thereby suggesting that clozapine is a promising and safe medication for suicidality [327]. However, treatment with clozapine poses detrimental side effects, including a substantial risk of agranulocytosis, which could lead to death [314].

### 13.2. Olanzapine

Olanzapine may be a competent maintenance drug treatment due to its antimanic and antidepressant effects [314]. In a group of patients with BD who were given olanzapine, a significantly better mean improvement in mania ratings and a significantly higher proportion of patients who attained remission were observed [314]. Furthermore, the results of an 8-week double-blind study of BD-I patients with depression administered placebo (*n* = 355) or olanzapine (5–20 mg/d; *n* = 351) revealed the efficacy of olanzapine as a therapeutic agent for mixed depression in BD-I [328].

### 13.3. Risperidone

Systematic investigations have demonstrated the efficacy and safety of using risperidone in the treatment of acute mania, either as an adjuvant therapy to lithium or valproate or as a stand-alone treatment [314]. In a 6-month multicenter open trial, risperidone proved to be safe and effective in the long term as an add-on treatment for TRBD, and it did not aggravate manic symptoms [314]. The study did reveal some adverse effects of risperidone, which were largely moderate and included weight gain [314]. A retrospective cohort study conducted with 469 BD-I patients who were given long-acting injections of risperidone for 1 year, in addition to concomitant BD medications, found that risperidone long-acting injections may decrease the severity of BD-I [157]. An exploratory analysis of 162 BD patients who had frequent relapses noted improvement in clinical status, depressive symptoms, and manic symptoms after add-on risperidone long-acting injection treatment [329].

Several other studies have investigated the efficacy of risperidone in pediatric patients with BD. One retrospective study observed pediatric patient charts over 18 months in an outpatient clinic for mood disorders [330]. Data obtained for BD patients with aggression who were prescribed risperidone revealed that mood stabilizers alone were ineffective in managing symptoms. Throughout the follow-up period, aggression and manic symptoms decreased in all patients [330]. Overall, the study established that the addition of risperidone may improve mania and aggression in pediatric BD patients who have inadequate responses to mood stabilizers alone [330]. A similar open study of 22 pediatric BD patients in a manic, hypomanic, or mixed state reported that 8 weeks of risperidone monotherapy (1.25 ± 1.5 mg/d) was associated with significantly improved symptoms [331].

### 13.4. Cariprazine

Cariprazine, which has been authorized for use in the treatment of mania accompanying BD, is a partial D_2_/D_3_ receptor agonist [332,333,334,335,336,337,338]. It appears to be a safe and effective medication for acute mania and mixed episodes in BD [339,340,341,342]. Some research data have indicated that both low and high doses of cariprazine are effective and well tolerated as a drug treatment for mania, depression, and psychosis [343,344,345]. RCTs of BD-I patients in manic and mixed episodes found the greatest treatment benefit when cariprazine was provided in the range of 3 to 12 mg [346,347]. Placebo-controlled studies of bipolar depression have indicated that 1.5 to 3 mg/d of cariprazine monotherapy is a competent treatment for acute depression in BD [346].

Durgam et al. conducted a phase II trial on 239 research participants assigned either to a placebo group or to a group receiving flexible doses of cariprazine [348]. Of the participants, 66.1% were administered a final dose of 12 mg/d, 16.9% were administered 9 mg/d, 12.7% were administered 6 mg/d, and 4.2% were administered 3 mg/d. Across the cariprazine group, the average dose was 8.8 mg/d [348]. Overall, the group administered cariprazine exhibited significantly greater gains in the Young Mania Rating Scale compared with the group that received placebo [348]. Another double-blind placebo-controlled trial randomly allocated placebo (*n* = 158), 1.5 mg/d of cariprazine (*n* = 157), and 3.0 mg/d of cariprazine (*n* = 165) in adult BD-I patients who were in a depressive episode [349]. Both cariprazine doses were safe, well tolerated, and effective in decreasing depressive symptoms [349]. Although a singular dosage recommendation has not been standardized for cariprazine yet, the results of these studies indicate its efficacy in certain cases of BD [346,347,348,349].

### 13.5. Quetiapine

The antipsychotic actions of quetiapine are likely derived from its antagonistic activity against DA D_2_ receptors and serotonin 5-hydroxytryptamine 2 receptors [350]. Quetiapine is an atypical antipsychotic agent that is administered orally [351,352]. On occasion, it is recommended for the treatment of BD, although some studies have suggested that it is more successful specifically in alleviating the symptoms of anxiety and depression accompanying BD [351,352,353,354]. Five double-blind RCTs evaluated the tolerability and effectiveness of quetiapine over 8 weeks in BD patients who were undergoing a major depressive episode [350]. Quetiapine monotherapy at 300 mg/d, that at 600 mg/d, and extended-release quetiapine monotherapy at 300 mg/d caused a significantly greater improvement in Montgomery–Asberg Depression Rating Scale scores compared with placebo [350]. Another RCT over 104 weeks found that quetiapine used as maintenance drug therapy was more effective than lithium or placebo in preventing relapse [350]. However, the study only included patients who previously responded to quetiapine during the acute phase of BD [350]. Thus, a bias for quetiapine over lithium in the study sample may have already been present. In the patients with bipolar depression, the adverse effects of quetiapine at 300 mg/d, quetiapine at 600 mg/d, and extended-release quetiapine at 300 mg/d were mild or moderate; in general, all three quetiapine doses were well tolerated [350].

## 14. Role of Therapies

A number of therapies have demonstrated successful outcomes in BD, including psychotherapies (CBT and ImCT), phototherapy (BLT), and ECT.

### 14.1. CBT

CBT has classically been used to treat depression and increase self-esteem through cognitive restructuring [355,356,357]. In addition to being used to treat unipolar depression, it has also been adapted to treat BD [358,359,360,361,362,363]. Studies on the use of CBT for BD have suggested the use of CBT as an add-on treatment to pharmacotherapy to prevent depressive symptoms and relapse [357,364]. Chiang et al. systematically analyzed the findings of 19 RCTs in which CBT was used as an adjuvant to pharmacotherapy [357]. Their analysis supported the adjunctive use of CBT in BD because of its clinical benefit both after treatment and during follow-up [357]. Moreover, a case study on 3 BD-II patients showed that the rate of BD recurrence could be decreased by the use of CBT as an adjuvant to medication [365]. The group format of CBT is also a potentially useful intervention for BD, as indicated by a study in which 41 patients with BD received 14 sessions of group CBT along with medication [366]. In the study, group CBT was found to improve depressive symptoms [366]. Likewise, another study of patients with BD reported that the group format of CBT was effective in decreasing fluctuations in mood state [356].

### 14.2. BLT

BLT, also known as phototherapy, uses glare therapy in the treatment of the symptoms of depression [367,368,369,370,371,372,373,374]. Some research studies have reported that BLT was successful as an adjunctive therapy for BD [375,376,377,378,379,380]. Tseng et al. demonstrated significant antidepressant activity with BLT [381]. Additional lines of evidence from RCTs have shown that BLT may reduce the symptoms of depression among the general population [382,383,384,385,386,387,388]. One benefit of BLT over antidepressants is that its risk of causing a switch to mania is lower (2.3% vs. 15–40%) [389]. Similarly, the effects of light therapy were analyzed in four trials on a total sample of 190 patients with bipolar depression, 94 of whom received the intervention [390]. The meta-analysis revealed a risk ratio of response to light therapy of 1.78 (95% confidence interval, 1.24–2.56) in patients with BD [390]. Another trial of 63 research participants investigated the effects of 1 h of light therapy daily for 2 weeks [391]. Thirty-three participants received BLT, whereas 30 received dim red light therapy. The outcomes of the study indicate that BLT may be an effective and safe adjuvant for acute depression in BD [391].

### 14.3. ImCT

An imagery-focused intervention addressing mood and anxiety in BD found that it helped reduce clinical symptoms [392,393]. In addition, patients with BD accepted and were highly satisfied with the intervention [392,393]. Eleven patients with BD were administered a combination of ImCT and standard care in one study, where their moods were monitored 6 months pre- and posttreatment [393]. In addition, their anxiety was measured for 1 month from the initiation of treatment. The study provided promising results, suggesting that the addition of ImCT to standard care could alleviate the depressive and anxious symptoms of BD in a manner that is satisfactory to patients [393].

### 14.4. ECT

ECT is a fast-acting and potent method for stimulating the brain [394,395]. It is often used in major depression, but it can also be effective in TRBD [396,397,398]. For instance, a study of 344 patients with BD found that ECT was safe and effective in all stages of severe and medication-resistant BD [399]. The quick antidepressant action of ECT likely contributes to long-term prevention of suicide in affective illnesses, such as BD and major depressive disorder [400,401]. An investigation of 487 patients with BD or unipolar depression from 2000 to 2013 reported that those who underwent ECT had a 19.7% lower probability of suicide compared with those who did not [401]. The therapeutic action of ECT for BD may be due to its influence on OS [396]. In one study, 28 TRBD patients and 49 controls received ECT, and several OS parameters were measured (SOD, GPX, CAT, and MDA) [396]. The outcomes suggested that a decline in lipid peroxidation levels contributed to the efficiency of ECT [396]. MDA levels were shown to decline exclusively in ECT responders, which points to the possible role of MDA reduction in the efficiency of ECT [396]. This suggests that OS is associated with BD severity and the response to ECT [396].

## 15. Conclusions

BD is becoming increasingly understood as a condition of aberrant neuroplasticity. Multiple factors, such as OS, imbalance of neurotransmitters, and genetics, are associated with the pathophysiology of BD. OS, caused by an imbalance between oxidant and antioxidant enzymes, may lead to cell damage. Decreased antioxidants and greater production of oxidizing agents lead to OS, causing alterations in proteins, carbohydrates, lipids, and DNA. In addition, reactive species act on mitochondria to eventually lead to increased concentrations of ROS/reactive nitrogen species, further oxidizing mitochondrial lipids, proteins, and DNA. In this regard, BD is associated with lipid peroxidation and DNA damage. Consistent with this observation, TBARS levels, which act as a lipid peroxidation marker, tend to be increased in patients with BD. Moreover, patients with BD have reduced BDNF levels, along with altered Ca^2+^ homeostasis and increased peripheral Ca^2+^ levels. Studies of both CNS-related and systemic cytokine changes in patients with BD have indicated that the neuroimmune system plays an important role in BD pathophysiology. Therefore, nutraceuticals may have a role in the adjuvant treatment of BD.

An accumulating body of evidence suggests that the therapeutic use of antioxidants in BD is beneficial in the treatment of depression associated with BD. Supplements that have been proposed to have therapeutic value in BD include vitamin D, omega-3 PUFAs, and folic acid. In addition to omega-3 fatty acids and vitamin D, other nutrients such as vitamin A, vitamin C, polyphenols, carotenoids, and other anti-inflammatory compounds might contribute to the homeostatic regulation of OS and inflammation. To date, lithium is the leading mood stabilizer used to ameliorate the pathophysiology of BD because of its effects on neuroplasticity. Oral antipsychotic agents, such as clozapine, olanzapine, risperidone, cariprazine, and quetiapine, are regarded as the first-line drug treatments for psychotic depression, and they are especially recommended for maintenance therapy. In addition, CBT, BLT, ImCT, and ECT have all been proven as effective adjuvant therapies for the treatment of BD.
